# Clinical efficacy of washed microbiota transplantation on metabolic syndrome and metabolic profile of donor outer membrane vesicles

**DOI:** 10.3389/fnut.2024.1465499

**Published:** 2024-11-19

**Authors:** Xuan Hu, Qingting Wu, Lingui Huang, Jiating Xu, Xingxiang He, Lei Wu

**Affiliations:** ^1^Department of Gastroenterology, Research Center for Engineering Techniques of Microbiota-Targeted Therapies of Guangdong Province, The First Affiliated Hospital of Guangdong Pharmaceutical University, Guangzhou, China; ^2^School of Biological Sciences and Engineering, South China University of Technology, Guangzhou, China

**Keywords:** washed microbiota transplantation, metabolic syndrome, clinical efficacy, outer membrane vesicles, metabolic profile

## Abstract

**Object:**

To clarify the clinical efficacy of washed microbiota transplantation (WMT) for metabolic syndrome (MetS), and explore the differences in the metabolic profile of bacterial outer membrane vesicles (OMVs) in donor fecal bacteria suspension received by MetS patients with good and poor outcomes, and to construct a predictive model for the efficacy of WMT for MetS using differential metabolites.

**Methods:**

Medical data 65 MetS patients who had completed at least 2 courses of WMT from 2017.05 to 2023.07 were collected. Fecal bacteria suspension of WMT donors were collected, and the clinical data of MetS patients treated with WMT during this period were collected as well. The changes of BMI, blood glucose, blood lipids, blood pressure and other indicators before and after WMT were compared. OMVs were isolated from donor fecal bacteria suspension and off-target metabolomic sequencing was performed by Liquid Chromatograph Mass Spectrometer (LC–MS).

**Results:**

Compared with baseline, Body mass index (BMI), Systolic blood pressure (SBP) and Diastolic blood pressure (DBP) of MetS patients showed significant decreases after the 1st (short-term) and 2nd (medium-term) courses, and fasting blood glucose (FBG) also showed significant decreases after the 1st session. There was a significant difference between the Marked Response OMVs and the Moderate Response OMVs. It was showed that 960 metabolites were significantly up-regulated in Marked Response OMVs and 439 metabolites that were significantly down-regulated. The ROC model suggested that 9-carboxymethoxymethylguanine, AUC = 0.8127, 95% CI [0.6885, 0.9369], was the most potent metabolite predicting the most available metabolite for efficacy.

**Conclusion:**

WMT had significant short-term and medium-term clinical efficacy in MetS. There were differences in the structure of metabolites between Marked Response OMVs and Moderate Response OMVs. The level of 9-Carboxy methoxy methylguanine in Marked Response OMVs can be a good predictor of the efficacy of WMT in the treatment of MetS.

## Introduction

Metabolic Syndrome (MetS) is a complex group of metabolic disorders including hypertension, dyslipidaemia, abdominal obesity and abnormal fasting blood glucose concentrations. MetS significantly increases a patient’s risk of diabetes, coronary heart disease, stroke, obstructive sleep apnoea, polycystic ovary syndrome and more. The effects of healthy gut microbiota on the human immune, metabolic, and digestive systems have been repeatedly studied in recent years (1, 2). Gut microbiota played an important role in maintaining health (3–5). Dysbiosis of the gut microbiota has been associated with the onset and progression of a variety of diseases (6–8). MetS patients suffer from disorders of gut microbiota, which are manifested by the proliferation of harmful bacteria and the inhibition of beneficial bacteria (9). In T2DM patients, the abundance of some metabolically beneficial microbiota, such as bacteria that produce butyrate, decreased, while pathogenic bacteria known to cause a variety of other conditions increased (9). Dyslipidemia can lead to an imbalance of gut microbiota, which in turn can further exacerbate the disorder of lipid metabolism (9). Fecal microbiota transplantation (FMT) is a therapeutic approach to treat a range of intestinal and extra-intestinal disorders by transplanting gut microbiota from the feaces of strictly screened human beings that meet the health criteria into the intestinal tract of patients to change the composition of the intestinal microbiota of the patients (10). Washed microbiota transplantation (WMT) is a microbiota transplantation method that is similar to traditional FMT but adds the safety measure of washed microbiota. The biggest difference between WMT and FMT is that the bacterial solution of WMT is prepared by an intelligent microorganism separation system (GenFMTer), which has gone through a multi-level filtration system, and finally the washed bacterial solution of WMT is obtained after several washed. It has better safety, quality control for bacterial flora disorders and effectiveness (11–13).

Wu et al. performed WMT on overweight patients and showed that WMT improved the gut microbiota of overweight patients compared to controls. At the genus level, the relative abundance of *Prevotella*, *Fusobacterium*, and *Enterococcus* was increased after WMT. The relative abundance of *Bacteroides*, *Escherichia-Shigella*, *Streptococcus*, and *Klebsiella* was reduced (14). Another clinical study also showed that in hyperglycaemic patients treated with WMT, WMT could reduce short-and mid-term fasting blood glucose levels in hyperglycaemic patients by modulating the gut microbiota, providing a new clinical pathway for the treatment of abnormal glucose metabolism (11). However, the clinical effect of WMT in the treatment of MetS is still unclear.

Bacterial outer membrane vesicles (OMVs) are nanoscale spherical structures produced by the outward budding of the outer membrane of Gram-negative bacteria, with diameters ranging from 20 to 250 nm, whose main components are proteins, nucleic acids, lipopolysaccharides, and enzymes (15). OMVs are important extra-bacterial communication mechanism, and in addition to its role in pathogenesis, stress response, OMVs play an important role in immunomodulation and the establishment and homeostasis of the gut microbiota (16, 17). A study by Engevik et al. found that the addition of purified OMVs to colonic epithelial cells stimulated the secretion of pro-inflammatory cytokines interleukin-8 (IL-8) and tumor necrosis factor (TNF) (18). Wang et al. found that transplantation of OMVs from the gut microbiota into the mouse intestine alleviated colitis and enhanced the therapeutic effect of programmed cell death protein (PD-1) immunotherapy against colorectal cancer (CRC) by maintaining intestinal homeostasis (19). OMVs have important physiological and pathological functions in the gut microbiota.

Studies have shown that the characteristics of the donor gut microbiota can influence the efficacy of WMT, such as the similarity of the gut microbiological characteristics of patients who have received transplants to those of donors (20), and the presence of specific microorganisms in the gut of the donor that can colonize the recipient (21). OMVs have an important mode of bacterial action in the host, the objective to clarify the clinical efficacy of WMT for MetS, and explore the differences in the metabolic profile of bacterial OMVs in donor fecal bacteria suspension received by MetS patients with good and poor outcomes.

## Materials and methods

### Patients and experimental design

Medical data, body mass index (BMI), blood pressure, fasting blood glucose (FBG), glycosylated hemoglobin (HbA1c), fasting insulin (FI), insulin resistance values (IRV), insulin resistance value (HOMA-IR), and total cholesterol (TC), Triglyceride (TG), Low-Density Lipoprotein Cholesterol (LDL-C), and High-Density Lipoprotein Cholesterol (HDL-C) were collected from MetS patients who attended The First Affiliated Hospital of Guangdong Pharmaceutical University, Guangzhou, China and completed at least 2 courses of WMT treatment from 2017.05 to 2023.07. The diagnosis of MetS in this study was as follows (22): patients were diagnosed with metabolic syndrome when 3 or more of the following were met: (1) Waist circumference: Male ≥90 cm, female ≥85 cm (or BMI ≥ 25.0 kg/m^2^, in China); (2) FPG ≥ 6.1 mmol/L or 2hPG ≥ 7.8 mmol/L and/or diagnosed with and treated for diabetes mellitus; (3) SBP/DBP ≥ 130/85 mmHg and/or (and/or diagnosed and treated for hypertension); (4) fasting blood TG ≥ 1.7 mmol/L; (5) fasting blood HDL-C < 1.04. Patients who had used antibiotic medication, who were taking probiotics during WMT treatment, and for whom the corresponding clinical information was severely missing were excluded. Based on the inclusion and exclusion criteria, a total of 65 patients with MetS were included. This study was conducted and approved by the Ethics Committee (No. 2021-13) in accordance with the Declaration of Helsinki at the First Affiliated Hospital of Guangdong Pharmaceutical University, Guangzhou, China. The participants provided their written informed consent to participate in this study.

### Procedure of WMT

Injections into the gastrointestinal (GI) tract are made through 2 routes: mid-gut transendoscopic enteral tubing (mid-gut TET) and colonic transendoscopic enteral tubing (colonic TET). The mid-gut TET included gastroscopic placement of a nasojejunal TET tube and freehand insertion of a nasojejunal tube; the colonic TET approach consisted of enteroscopic placement of an intestinal TET tube. In this study, the results of blood tests and other tests before the first course of treatment are the baseline values (WMT0), and relevant indicators will be obtained before each subsequent course of treatment. According to the standard treatment time of WMT, the “three-three course” was adopted. Time could be divided into short term (WMT1): about 1 month after the first WMT course; medium term (WMT2): about 2 months after the first WMT course; long term (WMT3): about 5 months after the first WMT course. Each course of treatment was injected continuously with a suspension of washed flora for 3 days, once a day, once a 120 mL of the washed bacteria solution was used to patients, and the total course of treatment lasted for a total of 5 months.

### OMVs collection and extraction

To prepare the washed microbiota, each 100 g of feces and 500 mL of 0.9% saline was used to prepare a homogeneous fecal suspension. Then, the washed bacteria solution was prepared by an intelligent microorganism separation system (GenFMTer) (one-hour FMT protocol with relatively low oxygen environment) (23). Fecal bacteria suspension from WMT donors were collected from which OMVs were isolated. Extraction of OMVs (24): During the preparation of fecal bacteria suspension from WMT, the supernatant of the fecal suspension was taken after the first centrifugation of the suspension for 3 min at 1,100 × g at room temperature, and then this fecal bacteria supernatant was centrifuged again for 30 min at 10,000 × g at 4°C, and the supernatant was collected by further removing impurities. The supernatant was further removed from impurities and collected. The supernatant after re-centrifugation was filtered twice through 0.45 μm pore size filters (Merck Millipore), 0.22 μm pore size filters (Merck Millipore) in that order. The sterile filtered supernatant was centrifuged at 100,000 × g for 2 h at 4°C. The precipitate was washed twice with 1 mL of sterile PBS and then suspended in 1 mL of sterile PBS. The precipitate was OMVs and stored in the refrigerator at −80°C. The OMVs were divided into marked response OMVs and moderate response OMVs by the 50% improvement rate of each clinical index collected above.

### The metabolic profile of OMVs by liquid chromatograph mass spectrometer

Three hundred microliter liquid sample was absorbed into 1.5 mL centrifuge tube, 400 μL extraction solution (acetonitrile: methanol = 1:1) was added, vortex mixed for 30 s, ultrasonically extracted at low temperature at 5°C and 40 KHz for 30 min, and the sample was left at −20°C for 30 min. Centrifuge at 4°C, 13,000 g centrifugal force for 15 min, remove the supernatant. After blowing dry with nitrogen, it was redissolved with 100 μL complex solution (acetonitrile: water = 1:1), ultrasonically extracted at low temperature for 5 min (5°C, 40KHz), and finally centrifuged at 4°C for 13,000 g for 5 min (25). The supernatant was transferred to the vial with internal cannula for machine analysis.

After the completion of the computer, LC–MS raw data was imported into the metabolomics processing software Progenesis QI (Waters Corporation, Milford, United States). At the same time will MS and MSMS mass spectrum information public databases and metabolic HMDB^1^ and Metlin,^2^ get metabolites information. Matrix data after searching the database was uploaded to the Majorbio Biocloud platform for data analysis.^3^ Firstly, data preprocessing was carried out. The data matrix retains at least one set of variables with non-zero values above 80%, and then filled the vacancy value (the minimum value in the original matrix fills the vacancy value). The response intensity of the sample essential spectrum peak was normalized by the sum normalization method, and the normalized data matrix was obtained to reduce the error caused by sample preparation and instrument instability. At the same time, variables with relative standard deviation (RSD) >30% of QC samples were deleted, and log10 logization was carried out to obtain the final data matrix for subsequent analysis.

### Statistical analysis

Data were analyzed using GraphPad Prism 8.0.2 and SPSS 27.0. For continuous variables, normality test was performed first, and mean ± standard deviation and one-sample *t*-test were used to describe and analyze continuous variables that conformed to normal distribution, respectively; and median and quartile, and one-sample rank sum test were used to describe and analyze continuous variables that did not conform to normal distribution, respectively. Frequency and percentile were used to describe categorical variables. Paired-sample *t*-tests were used for various physiological and biochemical indices of MetS patients before and after WMT that conformed to normal distribution, while rank-sum tests were used for those that did not conform to normal distribution, and one-way analysis of variance (ANOVA) was used for comparisons between multiple groups, with significant differences when the two-sided *p* < 0.05 was used.

## Results

### Clinical characteristics of patients with MetS undergoing WMT

Based on the inclusion and exclusion criteria, a total of 65 patients with MetS were included. The baseline levels of patients with MetS were shown in Table 1. Functional gastrointestinal disorder was the most important reason for performing WMT in 65 patients with MetS. Gastro-esophageal reflux disease (9, 13.85%), non-alcoholic fatty liver disease (8, 12.31%), gouty arthritis (4, 6.15%), pancreatitis (2, 3.08%), dermatitis (2, 3.08%), chemotherapy-related diarrhea (2, 3.08%), radiation proctitis (2, 3.08%), inflammatory bowel disease (2, 3.08%), hyperlipidaemia (1, 1.54%), epilepsy (1, 1.54%), hepatic encephalopathy (1, 1.54%), and depression (1, 1.54%) as shown in Table 2 below.

**Table 1:** Baseline in patients with MetS.

Variable	Baseline level (*n* = 65)
Age (y)	60.00 (50.00, 66.50)
Male (%)	58 (58.46)
Female (%)	42 (41.54)
BMI (kg/m^2^) (*n* = 64)	26.85 (24.89, 28.73)
FBG (mmol/L) (*n* = 65)	5.33 (4.75, 6.86)
HbA1c (%) (*n* = 27)	6.60 (5.90, 7.40)
FI (μU/mL) (*n* = 37)	13.24 (8.07, 19.01)
HOMA-IR (*n* = 37)	3.28 (2.24, 4.55)
TC (mmol/L) (*n* = 62)	4.81 (4.00, 5.82)
TG (mmol/L) (*n* = 62)	1.82 (1.25, 2.68)
LDL-C (mmol/L) (*n* = 62)	2.81 ± 1.05
HDL-C (mmol/L) (*n* = 62)	1.11 ± 0.29
SBP (mmHg)	131.88 ± 11.85
DBP (mmHg)	84.00 (76.00, 90.00)

BMI (kg/m^2^), Body mass index; FBG (mmol/L), Fasting blood glucose; HbA1c (%), Glycated hemoglobin; FI (μU/mL), Fasting insulin; HOMA-IR, Homeostasis model assessment of insulin resistance; TC (mmol/L), Total cholesterol; TG (mmol/L), Triglyceride; LDL-c (mmol/L), Low-density lipoprotein cholesterol; HDL-c (mmol/L), High-density lipoprotein cholesterol; SBP (mmHg), Systolic blood pressure; DBP (mmHg), Diastolic blood pressure.

**Table 2:** Primary diagnoses of patients treated with WMT.

Primary diagnosis	Number of examples	Percent (%)
Functional gastrointestinal disorders	30	46.15
Gastroesophageal reflux disease	9	13.85
Non-alcoholic fatty liver disease	8	12.31
Gouty arthritis	4	6.15
Pancreatitis	2	3.08
Dermatitis	2	3.08
Chemotherapy-associated diarrhea	2	3.08
Radiation proctitis	2	3.08
Inflammatory bowel disease	2	3.08
Hyperlipidemia	1	1.54
Epilepsy	1	1.54
Hepatic encephalopathy	1	1.54
Depression	1	1.54
Total	65	100%

### Effect of WMT on relevant clinical indicators in patients with MetS

Compared with the baseline, BMI, SBP, DBP of MetS patients showed significant decrease after the 1st and 2nd courses, and FBG also showed significant decrease after the 1st course, *p* < 0.05 was statistically significant. However, other indicators such as HbA1c (%), TC, TG, LDL-C, HDL-C, etc., *p* > 0.05 were not statistically significant. As shown in Tables 3, 4.

**Table 3:** Changes in relevant clinical indicators of MetS patients after 1 course of WMT.

	WMT0	WMT1	*p*-value
BMI (kg/m^2^)	26.86 (25.07, 28.73) (*n* = 63)	26.08 (23.88, 28.23) (*n* = 63)	0.002
FBG (mmol/L)	5.36 (4.76, 7.02) (*n* = 62)	5.01 (4.33, 6.28) (*n* = 62)	0.019
HbA1c (%)	6.87 ± 0.86 (*n* = 10)	6.86 ± 0.97 (*n* = 10)	0.945
FI (μU/mL)	15.05 ± 8.51 (*n* = 26)	15.04 ± 6.40 (*n* = 26)	0.999
HOMA-IR	3.27 (2.17, 4.86) (*n* = 26)	3.92 (2.03, 5.45) (*n* = 26)	0.790
TC (mmol/L)	5.25 ± 1.83 (*n* = 52)	5.05 ± 1.21 (*n* = 52)	0.427
TG (mmol/L)	1.95 (1.29, 2.71) (*n* = 52)	1.72 (1.25, 2.55) (*n* = 52)	0.071
LDL-C (mmol/L)	2.83 ± 1.02 (*n* = 52)	2.94 ± 1.00 (*n* = 52)	0.355
HDL-C (mmol/L)	1.14 ± 0.30 (*n* = 52)	1.17 ± 0.31 (*n* = 52)	0.340
SBP (mmHg)	131.88 ± 11.85 (*n* = 65)	127.69 ± 12.28 (*n* = 65)	0.033
DBP (mmHg)	82.86 ± 9.45 (*n* = 65)	79.37 ± 9.45 (*n* = 65)	0.019

BMI (kg/m^2^), Body mass index; FBG (mmol/L), Fasting blood glucose; HbA1c (%), Glycated hemoglobin; FI (μU/mL), Fasting insulin; HOMA-IR, Homeostasis model assessment of insulin resistance; TC (mmol/L), Total cholesterol; TG (mmol/L), Triglyceride; LDL-c (mmol/L), Low-density lipoprotein cholesterol; HDL-c (mmol/L), High-density lipoprotein cholesterol; SBP (mmHg), Systolic blood pressure; DBP (mmHg), Diastolic blood pressure.

**Table 4:** Changes in relevant clinical indicators of MetS patients after 2 courses of WMT.

	WMT0	WMT2	*p*-value
BMI (kg/m^2^)	27.32 ± 4.29 (*n* = 37)	26.54 ± 4.46 (*n* = 37)	0.033
FBG (mmol/L)	5.28 (4.74, 7.25) (*n* = 35)	4.95 (4.30, 6.59) (*n* = 35)	0.100
HbA1c (%)	7.77 ± 1.87 (*n* = 6)	7.87 ± 1.99 (*n* = 6)	0.567
FI (μU/mL)	14.44 ± 9.33 (*n* = 14)	15.25 ± 8.27 (*n* = 14)	0.626
HOMA-IR	2.91 (2.17, 4.86) (*n* = 14)	3.62 (1.95, 5.22) (*n* = 14)	0.875
TC (mmol/L)	4.96 ± 1.51 (*n* = 30)	4.94 ± 1.17 (*n* = 30)	0.934
TG (mmol/L)	1.97 (1.22, 2.66) (*n* = 30)	1.98 (1.34, 2.76) (*n* = 30)	0.497
LDL-C (mmol/L)	2.64 ± 1.07 (*n* = 30)	2.74 ± 1.04 (*n* = 30)	0.577
HDL-C (mmol/L)	1.15 ± 0.34 (*n* = 30)	1.13 ± 0.27 (*n* = 30)	0.639
SBP (mmHg)	130.97 ± 12.53 (*n* = 37)	125.68 ± 11.49 (*n* = 37)	0.047
DBP (mmHg)	81.78 ± 10.64 (*n* = 37)	79.05 ± 10.01 (*n* = 37)	0.197

BMI (kg/m^2^), Body mass index; FBG (mmol/L), Fasting blood glucose; HbA1c (%), Glycated hemoglobin; FI (μU/mL), Fasting insulin; HOMA-IR, Homeostasis model assessment of insulin resistance; TC (mmol/L), Total cholesterol; TG (mmol/L), Triglyceride; LDL-c (mmol/L), Low-density lipoprotein cholesterol; HDL-c (mmol/L), High-density lipoprotein cholesterol; SBP (mmHg), Systolic blood pressure; DBP (mmHg), Diastolic blood pressure.

### Effects of different TET placement methods on relevant clinical indicators of MetS patients and adverse reactions

The analysis found that the use of different GI tube placement methods for the first course of WMT did not have a significant effect on the relevant clinical indicators of MetS patients. As shown in Table 5.

**Table 5:** Effects of performing WMT using different TET placement methods on relevant clinical indicators of MetS patients.

	WMT1-WMT0 Mid-gut TET	WMT1-WMT0 Colonic TET	*p*-value
BMI (kg/m^2^)	−0.37 (−1.41, 0.27) (*n* = 24)	−0.39 (−1.46, 0.21) (*n* = 39)	0.465
FBG (mmol/L)	−0.23 (−1.00, 0.01) (*n* = 24)	−0.21 (−0.95, 0.35) (*n* = 38)	0.179
HbA1c (%)	/	/	/
FI (μU/mL)	1.02 ± 9.17 (*n* = 14)	−1.19 ± 5.61 (*n* = 12)	0.461
HOMA-IR	0.10 ± 3.11 (*n* = 14)	−0.50 ± 2.30 (*n* = 12)	0.608
TC (mmol/L)	0.17 ± 1.24 (*n* = 21)	−0.44 ± 2.01 (*n* = 31)	0.251
TG (mmol/L)	−0.35 ± 0.82 (*n* = 21)	−1.01 ± 4.07 (*n* = 31)	0.295
LDL-C (mmol/L)	0.25 ± 0.94 (*n* = 21)	0.02 ± 0.77 (*n* = 31)	0.784
HDL-C (mmol/L)	0.08 ± 0.30 (*n* = 21)	0.00 ± 0.18 (*n* = 31)	0.540
SBP (mmHg)	−5.68 ± 18.27 (*n* = 25)	−3.25 ± 13.64 (*n* = 40)	0.416
DBP (mmHg)	−3.28 ± 9.98 (*n* = 25)	−3.63 ± 12.48 (*n* = 40)	0.630

BMI (kg/m^2^), Body mass index; FBG (mmol/L), Fasting blood glucose; HbA1c (%), Glycated hemoglobin; FI (μU/mL), Fasting insulin; HOMA-IR, Homeostasis model assessment of insulin resistance; TC (mmol/L), Total cholesterol; TG (mmol/L), Triglyceride; LDL-c (mmol/L), Low-density lipoprotein cholesterol; HDL-c (mmol/L), High-density lipoprotein cholesterol; SBP (mmHg), Systolic blood pressure; DBP (mmHg), Diastolic blood pressure.

A total of 65 patients with MetS who had completed a cumulative total of 706 WMT sessions were included in this study. The incidence of adverse reactions was 3.5%, with diarrhea being the most common (10 cases, 1.4%), followed by abdominal pain (4 cases, 0.6%), nausea and vomiting (3 cases, 0.4%), generalized arthralgia (1 case, 0.1%), malaise (1 case, 0.1%), convulsions (1 case, 0.1%), rash (1 case, 0.1%), fever (3 cases, 0.4%) and dizziness (1 case 0.1%). However, these adverse reactions resolved within 24 h and did not pose an adverse effect on the patient’s health.

### Structural differences in OMVs metabolites between marked response OMVs and moderate response OMVs

**Figure 1:**
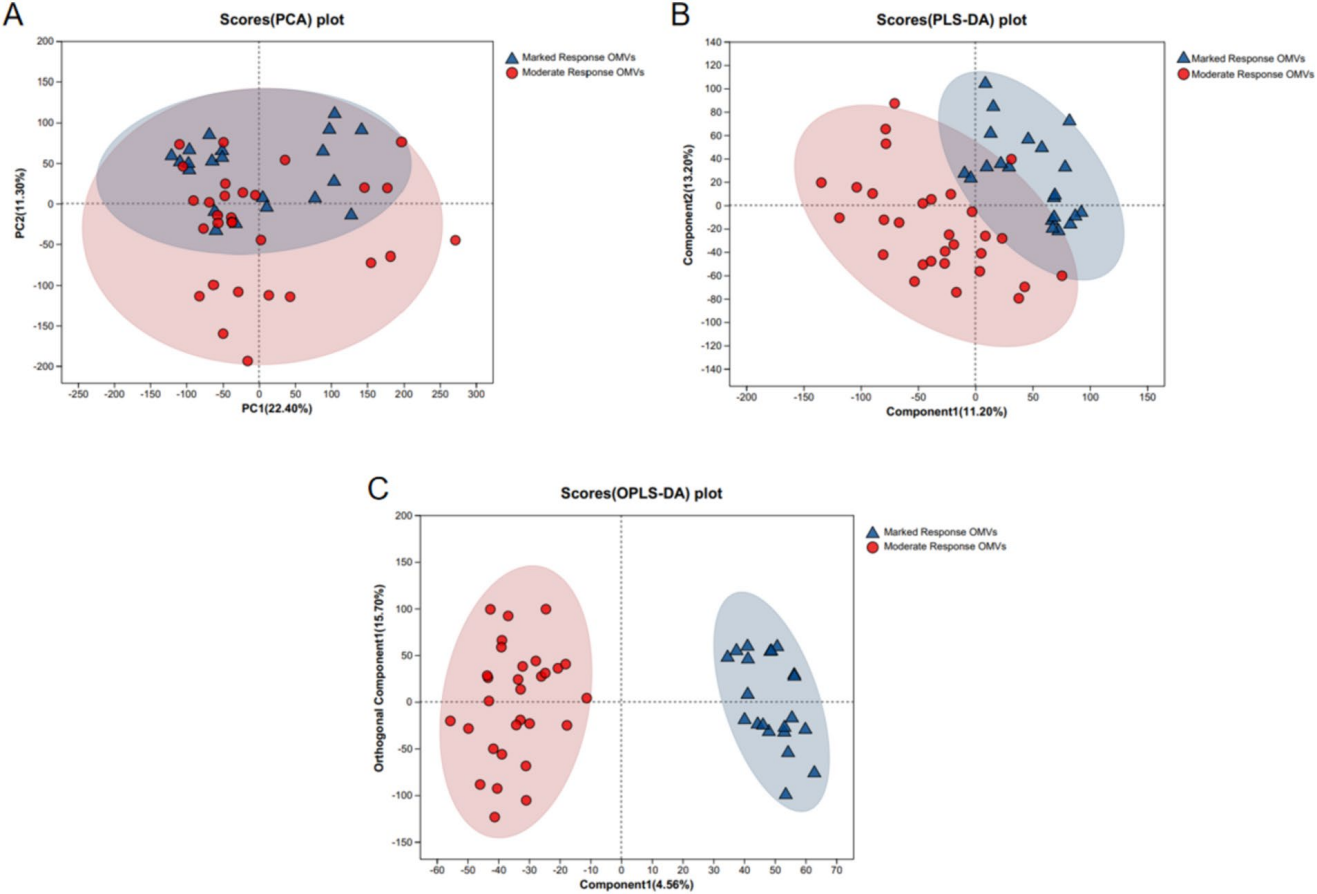
PCA and PLS-DA analysis of LC–MS metabolite profiles. (A) PCA analysis; (B) PLS-DA analysis; (C) OPLS-DA analysis. Marked response OMVs (*n* = 21) and Moderate response OMVs (*n* = 30).

PCA and PLS-DA models were applied to preliminarily analyze the differences between the quality donor group and the ordinary donor group and the magnitude of intra-group variability. It can be seen that the trend of separation between the two data groups is large, suggesting that there is a significant difference between the marked response OMVs and moderate response OMVs. As shown in Figure 1.

### Differences in OMVs-specific metabolite levels between marked response OMVs and moderate response OMVs

**Figure 2:**
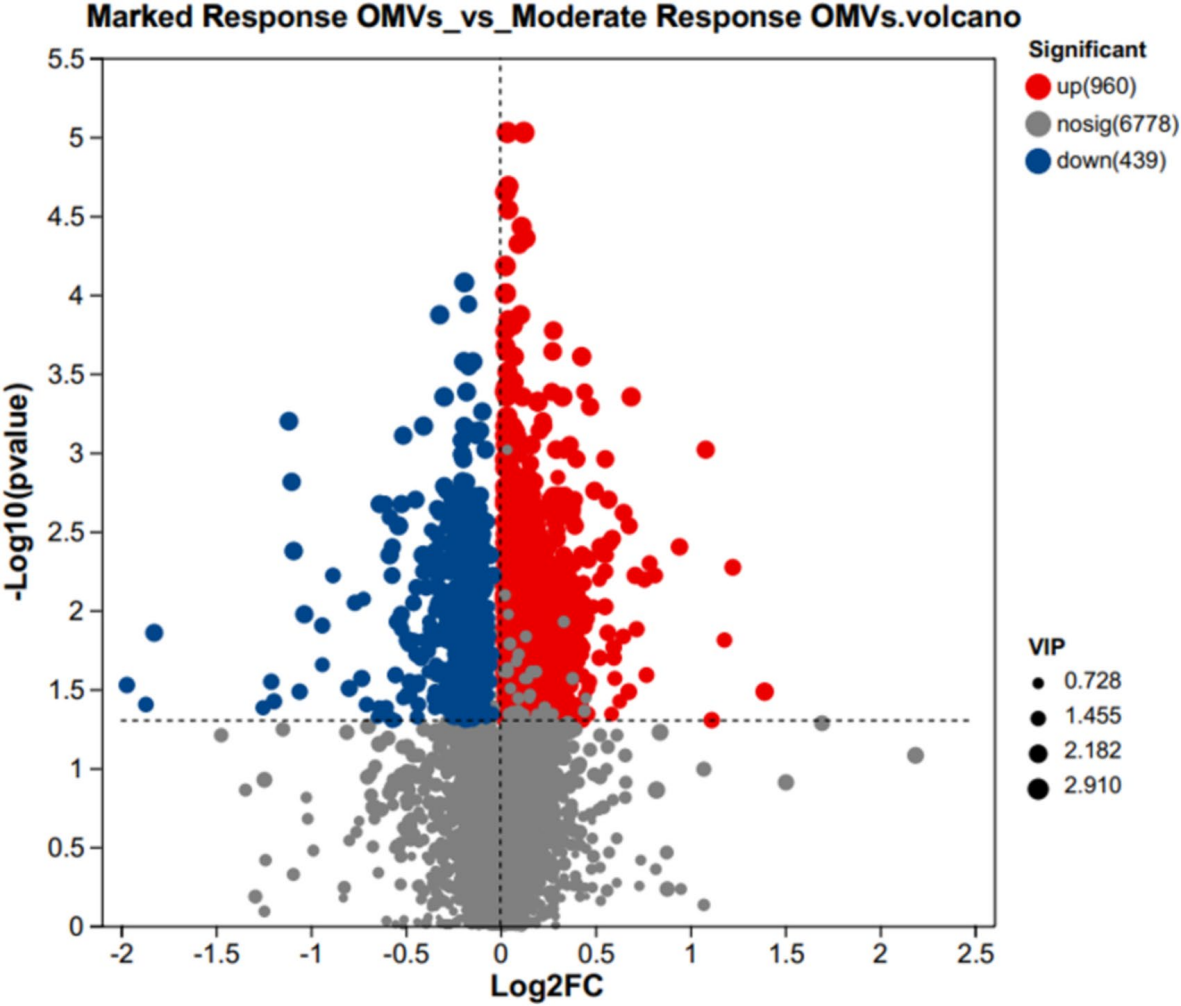
Volcanic maps of metabolites VIP values between marked response OMVs and moderate response OMVs. In red, 960 significantly up-regulated metabolites. In blue, 439 significantly down-regulated metabolites. Marked response OMVs (*n* = 21) and Moderate response OMVs (*n* = 30).

The volcano plot was used to screen out the differential metabolites between marked response OMVs and moderate response OMVs, when the metabolites were significantly different and highly expressed it is likely to mean that these metabolites are involved in important metabolic pathways. There were 960 metabolites that were significantly different and up-regulated in the premium donor group, and 439 metabolites that were significantly different and down-regulated (marked response OMVs/moderate response OMVs). There were 6,778 metabolites that were not significantly different. The volcano plot presents the trend of up-regulation and down-regulation of metabolites with differences between groups versus key metabolites with differences between groups. As shown in Figure 2.

**Figure 3:**
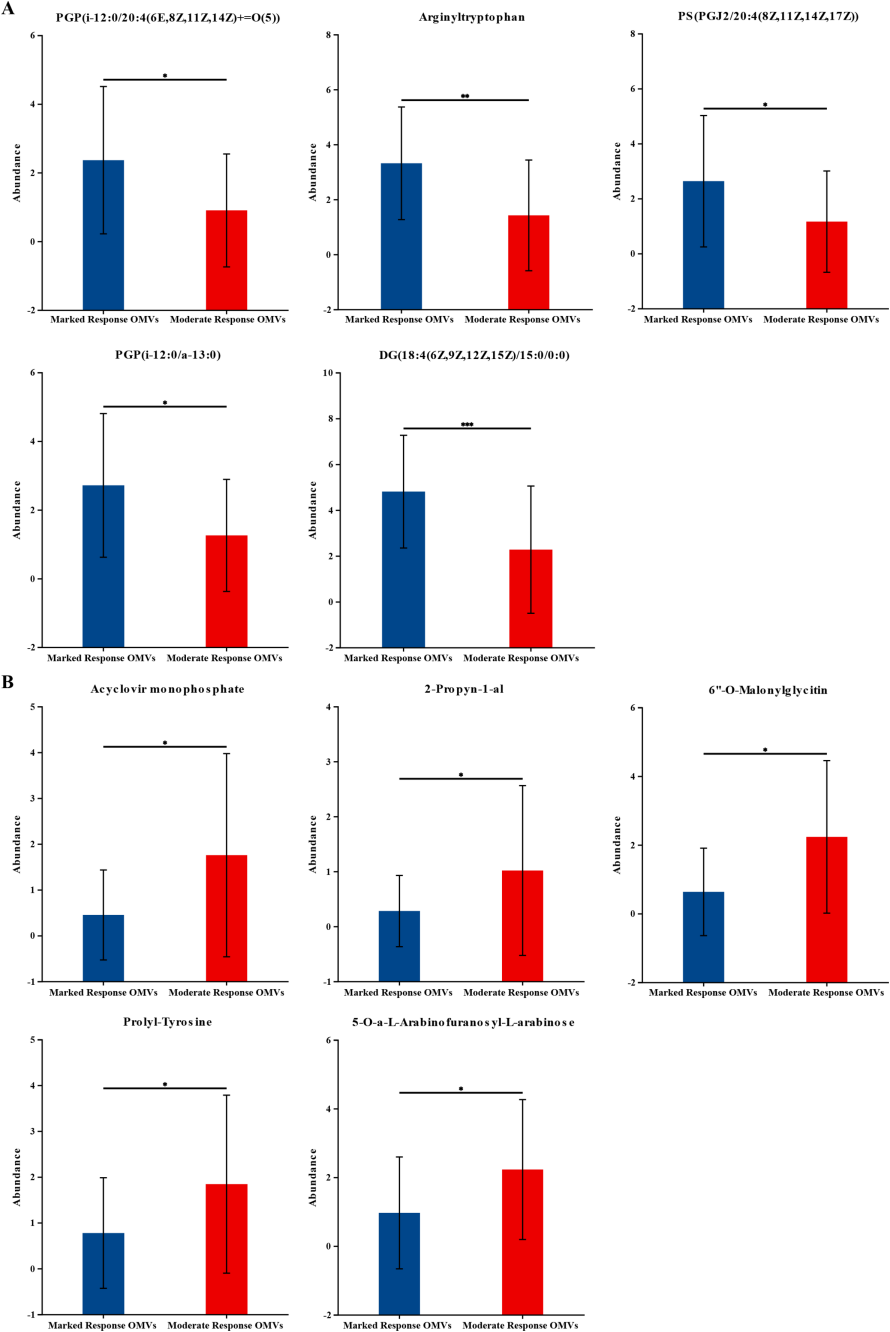
The levels of the significantly changed metabolites. (A) The top five of the 960 significantly up-regulated metabolites. (B) The top five of the 439 significantly down-regulated metabolites. The vertical coordinate (Abundance) is the mass spectrum intensity value (mass spectrum intensity after data preprocessing). Marked response OMVs (*n* = 21) and Moderate response OMVs (*n* = 30). * indicates 0.01 < *p* ≤ 0.05; ** indicates 0.001 < *p* ≤ 0.01. *** indicates *p* ≤ 0.001.

Subsequently, we further analyzed the metabolites that differed between groups. The top five of the 960 significantly up-regulated metabolites, ranked in order of fold difference, were PGP (i-12:0/20:4(6E,8Z,11Z,14Z) + =O(5)), Arginyltryptophan, PS (PGJ2/20:4(8Z,11Z,14Z,17Z)), PGP (i-12:0/a-13:0), DG (18:4(6Z,9Z,12Z,15Z)/15:0/0:0). The top five of the 439 significantly down-regulated metabolites in order of multiplicity of difference were Acyclovir monophosphate, 2-Propynyl-1-al, 6”-O-Malonylglycitin, Prolyl-Tyrosine, 5-O-a-L-Arabinofuranosyl-L-arabinose. As shown in Figure 3.

### ROC of OMVs metabolites in the MetS

**Figure 4:**
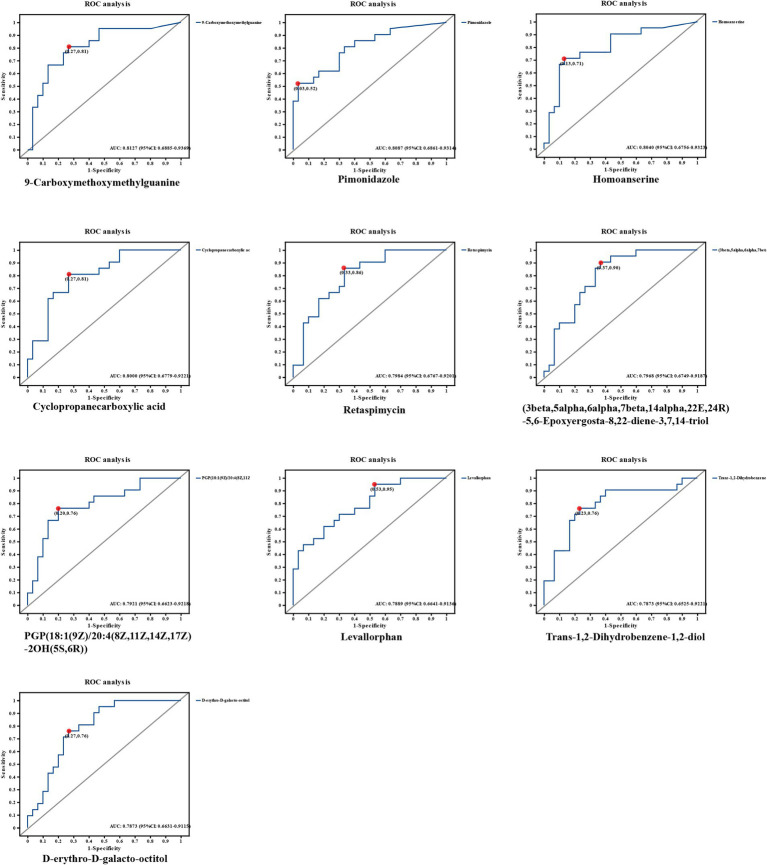
ROC graphs of metabolites with the highest AUC values. The top 10 metabolites based on the size of the AUC (area under the curve) were selected from a total of 1,399 significant metabolites. 0.1 < AUC < 1, the larger the value of AUC, the higher the prediction accuracy.

Next, we constructed the ROC model with the aim of evaluating the magnitude of the predictive effect of various metabolites on the efficacy of MetS, and the ROC model can visually present the metabolites that have a greater impact on efficacy. We ranked the top 10 metabolites based on the size of the AUC (area under the curve) were 9-Carboxymethoxymethylguanine, AUC = 0.8127, 95%CI [0.6885, 0.9369]; Pimonidazole, AUC = 0.8087, 95%CI [0.6861, 0.9314]; Homoanserine, AUC = 0.804, 95%CI [0.6756, 0.9323]; Cyclopropanecarboxylic acid, AUC = 0.8, 95%CI [0.6779, 0.9221]; Retaspimycin, AUC = 0.7984, 95%CI [0.6767, 0.9201], 3beta,5alpha,6alpha,7beta,14alpha,22E,24R-5,6-Epoxyergosta-8,22-diene-3,7,14-triol, AUC = 0.7968, 95%CI [0.6749, 0.9187]; PGP (18: 1(9Z)/20:4(8Z,11Z,14Z,17Z)-2OH(5S,6R)), AUC = 0.7921, 95%CI [0.6623, 0.9218]; Levallorphan, AUC = 0.7889, 95%CI [0.6641, 0.9136]; Trans-1,2-Dihydrobenzene-1,2-diol, AUC = 0.7873, 95%CI [0.6525, 0.9221]; D-erythro-D-galacto-octitol AUC = 0.7873, 95%CI [0.6631, 0.9115]. The metabolite 9-Carboxymethoxymethylguanine was the most available metabolite to predict efficacy. As shown in Figure 4.

## Discussion

In this study, we analyzed the clinical efficacy of WMT in the treatment of MetS and found that WMT significantly reduced the BMI, SBP, DBP, and fasting blood glucose levels of the patients at the same time, suggesting that WMT can improve patients with MetS. Our study demonstrated that a significant reduction in BMI was seen in the patients with MetS after WMT. It has been demonstrated that in mice, the gut microbiota can help the host to degrade carbohydrates in food (26) and participate in the process of energy absorption and fat storage (27, 28). Obesity has also been associated with dysbiosis of the gut microbiota as evidenced by a decrease in the diversity of gut bacteria in human studies (29, 30). FMT, as an emerging therapeutic approach, can restore the normal gut microbiota structure and function in patients (31). This suggested that WMT can treat obesity and reduce BMI by improving the intestinal flora of patients. Cani et al. found that mice ingesting probiotics may not only experience weight loss, but also improve insulin resistance in mice (32). Kootte et al. found that the improvement of insulin sensitivity in MetS patients in the lean donor group infused with lean donors after FMT may be related to the post-transplantation intestinal growth of the butyric acid-producing bacterium *Roseburia byresteria* growth (33). A prospective study observed that the gut microbiota of T2DM patients was reconfigured after FMT, as evidenced by higher levels of *Rikenellaceae* and *Anaerotruncus*, and significant decreases in patients’ HbA1c (%) and blood glucose levels (34). Similar to the results of the above mentioned study, our analysis suggests that WMT can significantly reduce blood glucose levels in patients with MetS by improving the gut microbiota of patients to significantly reduce blood glucose levels in MetS patients.

The present study also demonstrated that WMT can reduce blood pressure in hypertensive patients by improving the gut microbiota. Mell et al. demonstrated an association between gut microbiota and hypertension in rats (35). Adnan et al. also found that transplantation of microbiota from spontaneously hypertensive rats resulted in a significant increase in blood pressure in comparison to transplantation of gut microbiota from rats with normal blood pressure (36). In human studies, dysbiosis of the gut microbiota similarly contributes to the development of hypertension, and significant decreases in blood pressure have been observed in hypertensive patients after transplantation of their gut microbiota (37, 38). These remain consistent with the results of the present study. It is important to note that existing studies have shown significant decreases in lipid indices in both mice and humans after FMT (39, 40), where as our study demonstrated that WMT had no improvement in lipid indices in patients. By analyzing the baseline data, we found that in the baseline data of this study the baseline data of TC was described as 4.81 (4.00, 5.82) (mmol/L), TG was 1.82 (1.25, 2.68) (mmol/L), LDL-C was 2.81 ± 1.05 (mmol/L), and HDL-C was 1.11 ± 0.29 (mmol/L). The deviation of lipid levels from normal values before WMT in the MetS patients in this study was not significant, which may be the reason why the analyzes of the lipid indices in the MetS patients before and after WMT did not yield significant differences.

Different methods of tube placement contribute to differences in the disease outcome of FMT treatment (41–43). Compared with the baseline, BMI, SBP, DBP of MetS patients showed significant decrease after the 1st and 2nd courses, and FBG also showed significant decrease after the 1st course, *p* < 0.05 was statistically significant. However, other indicators such as HbA1c (%), TC, TG, LDL-C, HDL-C, etc., *p* > 0.05 were not statistically significant. In order to further investigate the influence of different catheterization methods on the clinical outcome of WMT, the analysis found that the use of different GI tube placement methods for the first course of WMT did not have a significant effect on the relevant clinical indicators of MetS patients. In this study, we compared the clinical indicators of MetS patients who completed the first course of WMT with two different tube placement methods, and no significant differences were found. This suggested that both middle gastrointestinal tube placement and lower gastrointestinal tube placement have an improvement effect on MetS. However, it should be noted that due to the small sample size of this study, the efficacy of each course of WMT could not be analyzed. A total of 65 patients with MetS who had completed a cumulative total of 706 WMT sessions were included in this study. The incidence of adverse reactions was 3.5%, with diarrhea being the most common (1.4%), followed by abdominal pain (0.6%), nausea and vomiting (0.4%), generalized arthralgia (0.1%), malaise (0.1%), convulsions (0.1%), rash (0.1%), fever (0.4%) and dizziness (0.1%). However, these adverse reactions resolved within 24 h and did not pose an adverse effect on the patient’s health. In addition, in this study, no serious adverse events occurred in all MetS patients who underwent WMT treatment, suggesting that WMT is not only effective but also safe for the treatment of MetS. Since adverse reactions are mainly observed at the time of WMT, further studies are needed for potential long-term adverse reactions or complications.

OMVs have different effects on the body depending on their composition. They can provide nutrients and digestive enzymes essential for metabolism and help repair the intestinal epithelial barrier. However, OMVs may also have deleterious effects, such as damaging intestinal epithelial cells, disrupting the intestinal epithelial barrier, and inducing intestinal epithelial cell death, including apoptosis, necrosis, autophagy, and other adverse effects (44–50). OMVs secreted by *Bacteroides fragilis* carries polysaccharide A (PSA), which is delivered to intestinal dendritic cells and induces CD4 regulatory T cells (Tregs) to produce IL-10, which down-regulates the inflammatory response and effectively ameliorates DSS-induced colitis (51–53). Harmful effects such as: delivery of virulence factors from *E. coli* OMVs to host intestinal macrophages, which upregulates the expression of pro-inflammatory cytokines such as IL-6 and TNF-*α*. This can lead to systemic inflammatory response syndrome (SIRS) and sepsis (54).

In addition, a role for OMVs in metabolic diseases has also been identified, and Seyama et al. demonstrated in mice that OMVs from *Porphyromonas gingivalis* reduces insulin sensitivity and contributes to the progression of diabetes by delivering gingival proteases to the liver (55). OMVs of the probiotic *Escherichia coli Nissle* 1917 (EcN) reduced body weight, lowered blood glucose, and increased plasma insulin levels in obese mice, and similarly EcN-OMVs modulated the intestinal microbiome in the intestinal tract, suggesting that EcN-OMVs may be able to regulate enterohepatic metabolism and ameliorate obesity and diabetes (56). OMVs are not only an important mode of bacterial action on the host, but also play an important role in metabolic diseases.

We observed differences in the structure of OMVs metabolites between the premium donor and regular donor groups by PCA and PLS-DA. The composition of metabolites in the premium donor group and regular donor group was further investigated with FC values of marked response OMVs/moderate response OMVs. We found 960 metabolites with significant differences and up-regulation. There were 439 metabolites that were significantly different and down-regulated, and the top five were Acyclovir monophosphate, 2-Propyn-1-al, 6″-O-Malonylglycitin, Prolyl-Tyrosine, 5-O-a-L-Arabinofuranosyl-L-arabinose. Acyclovir monophosphate, a metabolite of acyclovir, inhibits cellular DNA synthesis and kills infected cells (57). This follows the same trend as our metabolomics study, where we found significant downregulation of Acyclovir monophosphate in in common donors, which may partially explain the differences in donor efficacy. Xu et al. found that Prolyl-Tyrosine showed a significant positive correlation with *Collinsella* and *Coriobacteriaceae* (58). And whether the difference in the efficacy of WMT in MetS patients is related to these two genera needs to be further investigated. Subsequently, we constructed a ROC model to evaluate the magnitude of the predictive effect of various metabolites on the efficacy of MetS, and ranked the metabolites according to the magnitude of AUC, and the top one metabolites was: 9-Carboxymethoxymethylguanine, AUC = 0.8127, suggesting that it was good predictors of MetS efficacy. 9-carboxymethoxymethylguanine is a metabolite of the antiviral drug acyclovir in previous studies. Carboxymethoxymethylguanine has been suggested to be a metabolite of the antiviral drug acyclovir in previous studies and has been associated with impaired consciousness in “acyclovir encephalopathy” (59). Unita et al. investigated the effect of 9-carboxymethoxymethylguanine on plasma 9-carboxymethoxymethylguanine in patients with acyclovir encephalopathy, both before and after dialysis. Unita et al. measured plasma 9-carboxymethoxymethylguanine concentrations before and after dialysis in patients with acyclovir-associated encephalopathy, and concluded that a decrease in plasma 9-carboxymethoxymethylguanine levels may be one of the clinical biomarkers of consciousness in acyclovir-associated encephalopathy patients (60). In the present study, 9-Carboxymethoxymethylguanine could be used as a marker for the efficacy of WMT in the treatment of MetS, but the relationship between its mechanism of action and clinical efficacy needs further research.

This study has several limitations. First, this study mainly focused on the analysis of clinical index. The gut microbiota metagenomics and metabolomics before and after WMT have not been evaluated. Second, the impact of patient compliance resulting in a slightly small sample size, Therefore, more data are needed to confirm the clinical efficacy of WMT in the treatment of MetS. Third, we did not consider potential confounding factors between the main symptoms of WMT treatment and MetS. Although data show that WMT can improve MetS, we need large-scale prospective studies to further validate our conclusions. Fourth, although 9-carboxymethoxy-methylguanine can be used as a marker of efficacy in WMT treatment for MetS, its mechanism of action and its relationship to clinical efficacy need further investigation. In the future, we plan to conduct a large-sample prospective study to verify the effect of WMT on MetS.

## Conclusion

WMT had significant short-term and medium-term clinical efficacy in MetS. There were differences in the structure of metabolites between Marked Response OMVs and Moderate Response OMVs. The level of 9-Carboxy methoxy methylguanine in Marked Response OMVs can be a good predictor of the efficacy of WMT in the treatment of MetS.

## Data Availability

The original contributions presented in the study are included in the article/supplementary material, further inquiries can be directed to the corresponding authors.

## Footnotes

^1^ http://www.hmdb.ca/

^2^ https://metlin.scripps.edu/

^3^ https://cloud.majorbio.com
